# Supplementary material for: Development and validation of the dizziness fear-avoidance behaviours and beliefs inventory for patients with vestibular disorders
Source: PeerJ. 2023 Aug 29;11:e15940. doi: 10.7717/peerj.15940 (PMC10473040; doi:10.7717/peerj.15940)
Supplement: Supplemental Information 2 [file peerj-11-15940-s002.pdf]

CodeBook for the data base results:

Diagnosis:

1. Acute vestibular syndrome
2. Episodic vestibular syndrome
3. Chronic vestibular syndrome

Treatment received:
1. Yes
2. No

Treatment type:
1.Medical
2.Rehabilitation
3.Medical and rehabilitation
4. No treatment

BMI: Body mass Index

Gender:
1. men
2. woman

Education
1.uneducated
2.primary
3.secondary
4.university

Laboral
1.active
2.not employed
3. sick paid leave
4. retired

DHI_Total: Dizziness handicap Inventory
DHI_Functional Subscale
DHI_Emotional Subscale
DHI_Physical Subscale

HADS_TOTAL: Hospital Anxiety and Depression Scale.
HADS_A_TOTAL: Anxiety subscale
HAD_D_TOTAL: Depression subscale

D_FABBI_item number: Dizziness Fear-Avoidance Behaviors and Beliefs Inventory.

DHI_Clasi_Fun_Emoc: Result of the addition of the functional and emotional subscales.

CLASI_DHI: classification based on the addition of the DHI emotional and functional subscales:

1. No disability (0-14 points).
2. Moderate disability (15-24 points)
3. Sever disability (>25 points)

D_FABBI_TOTAL: Addition of all the items of the Dizziness Fear-Avoidance Behaviors and Beliefs Inventory

D_FABBI_Mov: Movement Fear-avoidance subscale results of the Dizziness Fear-Avoidance Behaviors and Beliefs Inventory

D_FABBI_ADL: Activities of daily living fear-avoidance subscale results of the Dizziness Fear-Avoidance Behaviors and Beliefs Inventory.
