# Supplementary material for: Development and validation of the dizziness fear-avoidance behaviours and beliefs inventory for patients with vestibular disorders
Source: PeerJ. 2023 Aug 29;11:e15940. doi: 10.7717/peerj.15940 (PMC10473040; doi:10.7717/peerj.15940)
Supplement: Supplemental Information 3 [file peerj-11-15940-s003.pdf]

Cuestionario de Creencias y Conductas de Miedo y Evitación al Mareo. CCMEM

Por favor, señale la respuesta más apropiada a las conductas y pensamientos asociados a la percepción de mareo o vértigo.

	Totalmente desacuerdo	Algo en desacuerdo	Algo de acuerdo	Totalmente de acuerdo
1. Siempre pienso en el movimiento que voy a hacer antes de realizarlo físicamente.	☐	☐	☐	☐
2. Tengo miedo a mover el cuello y la cabeza	☐	☐	☐	☐
3. Evito girarme en la cama para que no aparezcan los síntomas	☐	☐	☐	☐
4. No es seguro que una persona con mi problema camine sola por la calle.	☐	☐	☐	☐
5. Intento caminar con pasos cortos y lentos.	☐	☐	☐	☐
6. Tengo miedo a caerme.	☐	☐	☐	☐
7. Tengo miedo a conducir vehículos.	☐	☐	☐	☐
8. Evito realizar actividad física y deportiva.	☐	☐	☐	☐
9. Evito subir alturas como escaleras o balcones por mi problema.	☐	☐	☐	☐
10. Evito utilizar el ordenador o la tablet.	☐	☐	☐	☐
11. Una persona con mi problema no debería realizar ejercicio	☐	☐	☐	☐
12. Evito realizar algunas actividades domésticas	☐	☐	☐	☐
13. Tengo miedo de agacharme	☐	☐	☐	☐
14. Me produce malestar mirar hacia arriba por eso lo evito.	☐	☐	☐	☐
15. Intento evitar girar la cabeza para mirar a un lado	☐	☐	☐	☐
16. El mareo me indica que no debo moverme	☐	☐	☐	☐
17. Cuando salgo a caminar intento desplazarme por lugares en donde pueda sujetarme para ir más seguro.	☐	☐	☐	☐
