# Supplementary material for: Development and validation of the dizziness fear-avoidance behaviours and beliefs inventory for patients with vestibular disorders
Source: PeerJ. 2023 Aug 29;11:e15940. doi: 10.7717/peerj.15940 (PMC10473040; doi:10.7717/peerj.15940)
Supplement: Supplemental Information 4 [file peerj-11-15940-s004.pdf]

Dizziness Fear-Avoidance Behaviors and Beliefs Inventory

Please, indicate the most appropriate response to your beliefs and behaviors associated with your perception of dizziness or vertigo.

	Total disagreement	Some disagreement	Some agreement	Totally agree
1. I always think about the movement I'm going to make before I physically do it.	☐	☐	☐	☐
2. I'm afraid to move my neck and head.	☐	☐	☐	☐
3. I'm afraid of falling down.	☐	☐	☐	☐
4. It's not safe for someone with my condition to walk the street alone.	☐	☐	☐	☐
5. I try to walk with short, slow steps.	☐	☐	☐	☐
6. I avoid turning over in bed so as to prevent the symptoms from appearing.	☐	☐	☐	☐
7. I'm afraid to drive.	☐	☐	☐	☐
8. I avoid physical and sports activity.	☐	☐	☐	☐
9. Because of my condition, I avoid climbing heights such as stairs and balconies.	☐	☐	☐	☐
10. I avoid using computers and tablets.	☐	☐	☐	☐
11. A person with my condition should not exercise.	☐	☐	☐	☐
12. . I avoid household activities.	☐	☐	☐	☐
13. I'm afraid to bend down.	☐	☐	☐	☐
14. I avoid looking up because it makes me feel sick.	☐	☐	☐	☐
15. I avoid turning my head to look to the side.	☐	☐	☐	☐
16. The dizziness tells me that I shouldn't move.	☐	☐	☐	☐
17. When I go out for a walk, I try to walk in places where I can hold on to something to walk more safely.	☐	☐	☐	☐
